# Cytokine Levels in Saliva Are Associated with Salivary Gland Fibrosis and Hyposalivation in Mice after Fractionated Radiotherapy of the Head and Neck

**DOI:** 10.3390/ijms242015218

**Published:** 2023-10-16

**Authors:** Olga Zlygosteva, Inga Solgård Juvkam, Hans Christian D. Aass, Hilde K. Galtung, Tine M. Søland, Eirik Malinen, Nina F. J. Edin

**Affiliations:** 1 Department of Physics, University of Oslo, 0371 Oslo, Norway; olga.zlygosteva@fys.uio.no (O.Z.); eirik.malinen@fys.uio.no (E.M.); 2 Institute of Oral Biology, University of Oslo, 0372 Oslo, Norway; i.s.juvkam@odont.uio.no (I.S.J.); hilde.galtung@odont.uio.no (H.K.G.); t.m.soland@odont.uio.no (T.M.S.); 3 The Blood Cell Research Group, Department of Medical Biochemistry, Oslo University Hospital, 0450 Oslo, Norway; h.c.aass@medisin.uio.no; 4 Department of Pathology, Oslo University Hospital, 0372 Oslo, Norway; 5 Department of Radiation Biology, Oslo University Hospital, 0379 Oslo, Norway

**Keywords:** cytokines, X-rays, fractionated radiotherapy, normal tissue response, head and neck, salivary glands, fibrosis, mice

## Abstract

Cytokines are mediators of inflammation that could lead to fibrosis. The aim was to monitor cytokine levels in saliva and serum after locally fractionated radiotherapy of the head and neck in mice and investigate associations with salivary gland fibrosis and hyposalivation. C57BL/6 mice were randomized to sham or X-ray irradiation of 66 Gy in 10 fractions over 5 days. Blood and saliva were collected on days −7, 5, 35, 80, and 105 following cytokine analysis. The harvested submandibular salivary gland was assessed for the presence of fibrosis. Decision tree regression analysis was used to investigate whether cytokine levels could predict late endpoints in terms of hyposalivation or fibrosis. Significant formation of fibrosis in gland tissue and reduced saliva production was found after irradiation. The pro-inflammatory cytokines IL-1α, TNF, TIMP1, G-CSF, KC, and MIP-1α showed increased levels in saliva in irradiated mice and a strong correlation with late endpoints. The decision tree analysis largely separated controls from irradiated animals, with IL-1α being the strongest predictor. Pro-inflammatory cytokines in saliva, but not in serum, were associated with late endpoints. This indicates that cytokine expression in saliva is a good biomarker for local salivary gland damage with IL-1α as the strongest single predictor.

## 1. Introduction

More than 60% of head and neck (H&N) cancer patients experience salivary gland hypofunction after radiotherapy (RT) [1]. In addition, RT can result in complications such as mucositis, chewing impairment, and oral infections. In general, this may lead to late effects such as pain, dysphagia, and decreased sense of taste, all of which substantially reduce quality of life [2]. Ionizing radiation triggers biological responses in malignant tumors as well as in normal tissues, which can influence the secretion of cytokines and growth factors. These molecules are involved in several processes such as cell–cell signaling and immunomodulation [3,4,5] and can function in a paracrine, autocrine, or endocrine manner [6,7]. Cytokines have been found to induce both local and systemic responses and are important mediators of both acute and chronic inflammation in normal tissue after irradiation [8]. Chronic inflammation is characterized by coexisting inflammation, tissue injury, and fibrosis [9]. In irradiated tissues, fibrosis is consequential late tissue damage [10,11,12] and cytokines may play an important role in its development and manifestation. Therefore, cytokines could potentially be used as diagnostic biomarkers for tissue injuries following RT. Furthermore, they might function as therapeutic targets, or be used as monotherapy or a booster for other therapeutic agents to reduce radiation-induced tissue damage [7].

Modern laboratory techniques allow for measuring cytokines in tissues and various biological fluids at mRNA or protein levels [13]. As cytokines induced by RT are believed to be tissue-specific, several groups have studied various pro-inflammatory (IL-1α, IL-2, IL-6, TNF, IFN-γ), pro-fibrotic (TGF-β1), and stem-cell mobilizing (GM-CSF) cytokines in tissues and their correlation with cytokines in blood [14,15]. Biological fluids are generally more straightforward to collect than tissue biopsies and can be repeatedly sampled for screening. However, cytokine levels in fluids should correlate with relevant tissue processes or clinical endpoints in order to be used as surrogate biomarkers [16]. Some clinical and preclinical studies have shown changes in cytokine profiles in blood (serum or plasma) after total body irradiation or local radiation treatment [17,18,19,20]. However, only limited data exist on the association between cytokine levels in other biological fluids and clinical endpoints.

For local H&N irradiation, only a small number of clinical and preclinical studies have demonstrated elevated cytokine levels in blood and associations with inflammation and radiation-induced effects, especially salivary gland dysfunction [21,22]. Some clinical studies have reported dysregulated cytokine networks in saliva from H&N cancer patients after RT or in patients with hyposalivation as in Sjögren’s syndrome [23,24,25,26,27,28,29,30]. To our knowledge, no studies have monitored cytokine expression in both saliva and blood after irradiation of the H&N region and compared their level of association with late endpoints. Here, we hypothesize that cytokines in saliva are more specific for salivary gland effects after local RT than those in blood. This is because the cytokine background in blood, which reflects normal physiological processes, is expected to dominate over a possible radiation-induced elevation of a limited set of cytokines released from a small irradiated area. Thus, salivary cytokines could be more precise and reliable biomarkers for salivary gland effects after H&N irradiation. Additionally, saliva would be advantageous in screening for irradiation effects due to reduced invasiveness, simplified logistics in sample collection, and higher acceptance by participants in potential clinical trials [31].

The aim of this work was to monitor salivary cytokine levels after local fractionated irradiation of the H&N area in mice and to compare these with cytokines in serum. Furthermore, we aimed to correlate the cytokines with late irradiation effects in salivary glands and to investigate the cytokine profiles as potential biomarkers of late effects in salivary glands.

## 2. Results

### 2.1. Cytokine Expression

**Figure 1:**
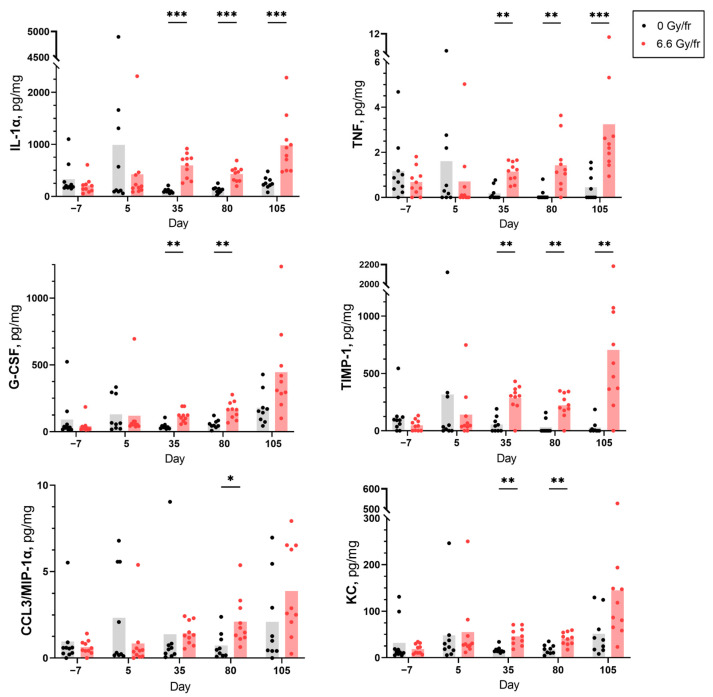
Levels of salivary cytokines IL-1α, TNF, G-CSF, TIMP-1, MIP-1α, and KC before and after fractionated irradiation at days −7, 5, 35, 80, and 105 (*n* = 9 in the control group, *n* = 10 in the irradiated group). Each dot represents an individual mouse. Data are presented as mean pg/mL cytokine per total protein mg/mL (* *p* < 0.05, ** *p* < 0.01, *** *p* < 0.001).

From the panel of 12 cytokines, 6 were detected at measurable levels in the saliva and serum samples from both irradiated and control groups. The levels of the detected cytokines in saliva at different time points are presented in Figure 1, and all these cytokines showed differences between the irradiated and control groups. Typically, the first time point showing a significant increase in salivary cytokine levels for irradiated animals was day 35. The increase in levels was significant for IL-1α, TNF, and TIMP-1 in irradiated mice compared to controls on days 35, 80, and 105 after the onset of irradiation. The levels of G-CSF and KC were only significantly higher in the irradiated group on days 35 and 80, while an increase in MIP-1α was only observed on day 80. In contrast, there was a tendency towards a decrease in IL-1α, TNF, MIP-1α, and TIMP-1 levels in saliva from irradiated mice sampled on day 5 (24 h after the last fraction). In serum, only MIP-1α was significantly decreased in the irradiated mice on days 35, 80, and 105 compared to controls, while the rest of the cytokines did not show any significant changes (Figure S1 in Supplementary Materials).

### 2.2. Fibrosis and Saliva Volume

**Figure 2:**
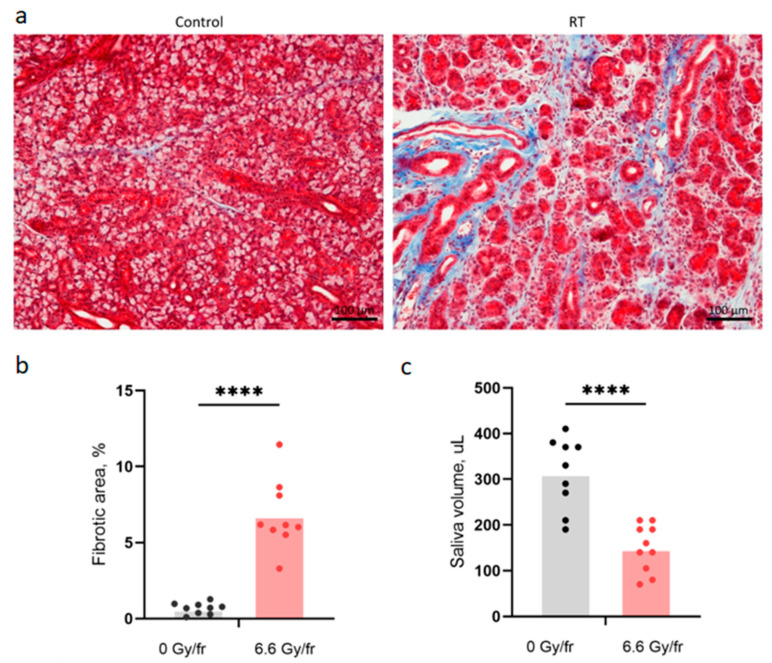
(**a**) Images of Masson Trichrome stained sections of the submandibular gland (SMG) in control and irradiated mice on day 105 with fibrotic regions in blue. (**b**) Fibrotic area fraction in SMG. (**c**) Pooled saliva volume. Each dot represents an individual mouse. Scale bar is 100 µm (**** *p* < 0.0001).

The fraction of fibrotic area in the SMG and the saliva volume, as a measure of salivary gland function, were used as late endpoints (Figure 2). Increased connective tissue around some large SMG ducts and replacement of acinar cells by connective tissue were seen in histological sections (Figure 2a). The fraction of fibrotic area in the SMG was significantly higher in the irradiated mice compared to controls (Figure 2b). The irradiated mice also showed significantly decreased saliva production (Figure 2c).

### 2.3. Correlations between Cytokines and Endpoints

**Figure 3:**
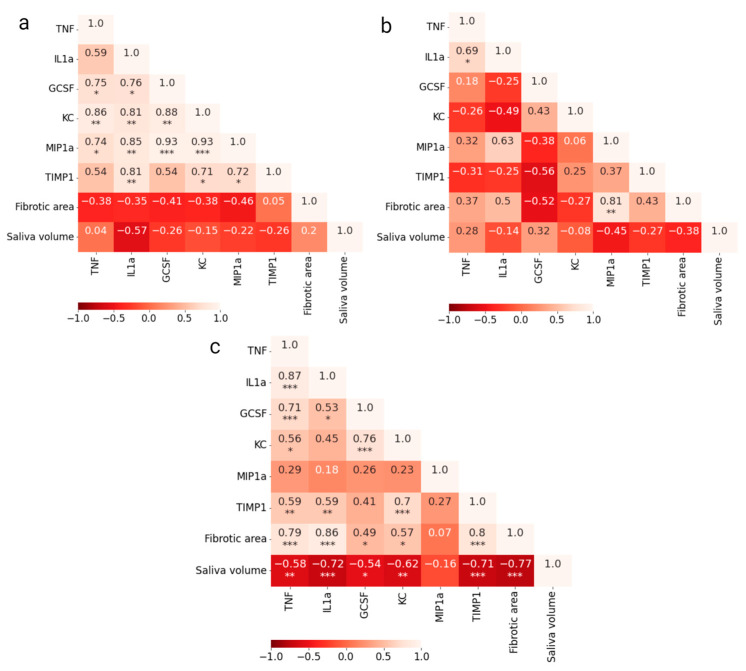
Pearson’s correlation matrix with significance for salivary cytokine levels at day 35 from (**a**) control mice; (**b**) irradiated mice; (**c**) both combined (* *p* < 0.05, ** *p* < 0.01, *** *p* < 0.001).

Pearson’s correlation between cytokine levels and endpoints (fibrotic area and saliva volume) for both groups was calculated for each time point. Correlation matrices for salivary cytokines at day 35 are shown in Figure 3a,b (correlation matrices for other time points are given in Figure S2 in the Supplementary Materials). At day 35, all cytokines in the control group were positively correlated to each other, while the cytokine levels in the irradiated group seemed to be more independent or weakly correlated. In the correlation matrix combining data from irradiated and control mice, a positive correlation of salivary cytokines with fibrotic area and a negative correlation with saliva volume was found (Figure 3c). For cytokines in serum, correlation matrix is given in Figure S3 in the Supplementary Materials. These correlations are generally lower than for cytokines in saliva.

**Figure 4:**
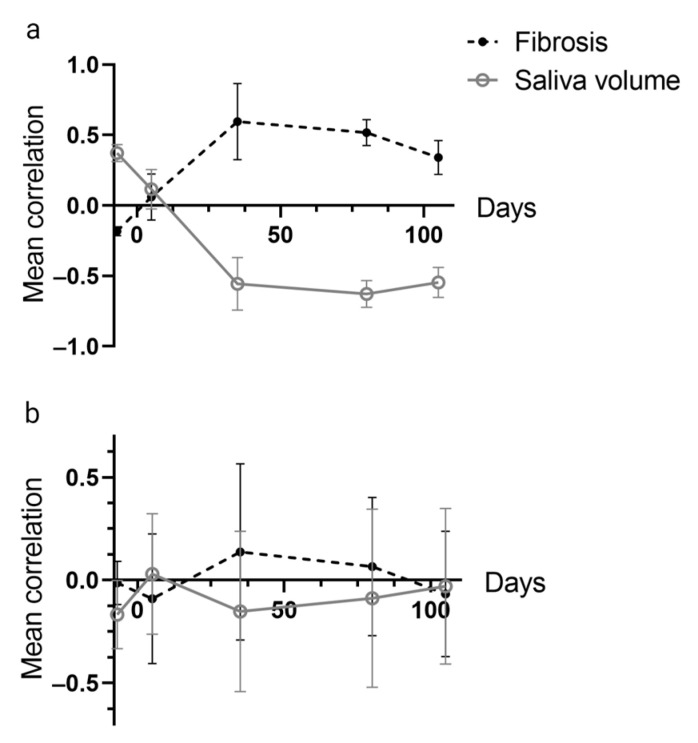
Mean correlation of (**a**) salivary and (**b**) serum cytokines with endpoints (fibrosis and saliva volume) at all time points.

The mean correlation of the whole cytokine panel with saliva volume and fibrosis for different time points is shown for salivary cytokines (Figure 4a) and serum cytokines (Figure 4b), respectively. As seen, the correlation between cytokines and endpoints is maximal (r = 0.6) around day 35. Moreover, correlation curves for fibrosis and hyposalivation are mirror images of each other, reflecting the difference in scoring (high and low levels indicate greater severity for respective endpoints) and that the endpoints are related. Importantly, there was a much stronger correlation between the salivary cytokines and endpoints than between the serum cytokines and endpoints.

### 2.4. Prediction of Late Effects Based on Early Cytokine Levels

**Figure 5:**
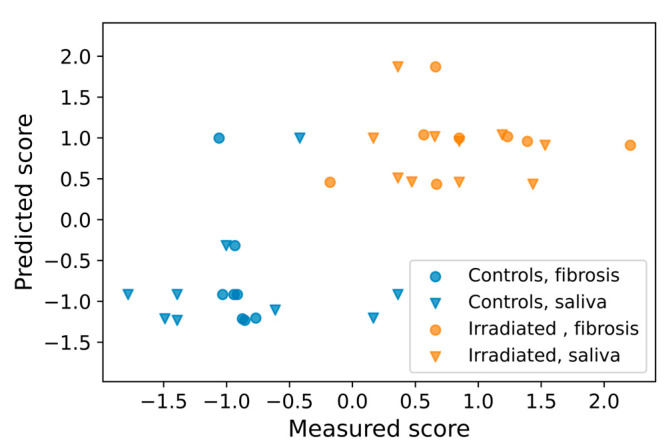
The decision tree prediction model for fibrosis and saliva volume, where the measured score is plotted against the predicted score.

Several of the salivary cytokines were associated with the two endpoints; fibrotic area and saliva volume. The decision tree regression model used all cytokine levels at a given time to predict the endpoints in a leave-one-out-cross-validation procedure. In a predictive assay, early assessment of predictors is pivotal, but cytokine levels at day −7 and day 5 showed no correlation with the endpoints (Figure 4). Thus, cytokines at day 35 were selected for the analysis. In Figure 5, the measured standard score (representing fibrotic area and saliva volume) is plotted against the predicted score. As seen, controls and irradiated animals are largely separated by the prediction model. The RMSE was in this case 0.75, compared to a baseline RMSE of 1.05 for a model where the mean measured overall score was used as the predictor. Separating the RMSEs for fibrotic area and saliva volume gave 0.76 and 0.8, respectively. The prediction using cytokine levels at day 80 or 105 was less good

The importance of each cytokine to the prediction model was 0.05, 16.5, 1.02, 1.50, 1.61, and 1.42 (relative units) for TNF, IL-1α, GC-SF, KC, MIP-1α, and TIMP1, respectively, indicating that IL-1α is by far the strongest contributor to the prediction model.

## 3. Discussion

Irradiation initiates rapid molecular and cellular responses, which include activation of various repair and survival signaling pathways and cytokine secretion, leading to an acute inflammatory reaction in the affected tissues. In addition, radiation-induced changes in gene expression and dysregulated cytokine expression contribute to late tissue responses such as chronic inflammation and fibrosis [15,20]. Most of the cytokines have overlapping functions and can also recruit and activate other cytokines or cell types involved in the fibrotic process, which enhances and complicates the response [14,20].

In order to use cytokines as predictive biomarkers of late effects, it is important to appraise when and where the cytokines are released subsequent to irradiation. We monitored cytokine release into saliva as a function of time after irradiation and correlated the cytokine expression data to two late endpoints; saliva volume and salivary gland fibrosis. In addition, the experiments were designed specifically for studying relevant normal tissue effects after H&N RT using local field compared to other preclinical studies using total body irradiation. The latter might result in lower cytokine secretion, especially in the blood, which makes our approach more clinically relevant.

To our knowledge, this is the first study of changes in cytokine levels in mouse saliva after irradiation. Interestingly, the salivary cytokine levels in the first 24 h after the last fraction were not significantly changed. However, we found an increase in the levels of six salivary cytokines on day 35, and a further increase on days 80 and 105. This is in line with other studies showing persistent waves of cytokine secretion in lung tissue or blood for a prolonged time after partial irradiation [16]. The temporal pattern of cytokine secretion may be explained by the reactivate responses in the tissues to deal with induced damage [3]. For the potential use of cytokine levels as biomarkers for late effects, the early time points for sampling are most relevant (well before the clinical manifestation of endpoints) and we have, therefore, focused on the salivary cytokine data from day 35 in the correlation and prediction models.

The formation of fibrosis in SMG tissue as well as reduced saliva production were used as endpoints for late effects after irradiation. The SMG was chosen for the assessment of fibrosis because it is the largest salivary gland in mice and, therefore, is easier to dissect in one piece for scoring the irradiated area. The early phases of radiation-induced fibrogenesis are similar to those of wound healing. However, while wound healing involves very early secretion of pro-inflammatory cytokines in blood or irradiated tissue [16,32], our experiments only showed increased levels of the investigated cytokines IL-1α, TNF, TIMP1, G-CSF, KC, and MIP-1α in the saliva from irradiated mice after day 35. All these cytokines have been reported to be highly involved in inflammatory responses and fibrosis formation, supporting the theory of cytokine-regulated development of radiation-induced late effects. For instance, IL-1α and TNF may activate the inflammatory process after irradiation and contribute to fibrosis under chronic inflammation by stimulation of fibroblast proliferation [8,33]. IL-1 is also considered a pro-inflammatory senescence-associated secretory phenotype (SASP) factor [34]. A recent mouse study demonstrated that radiation-induced cellular senescence in the salivary gland stem/progenitor cell (SGSC) niche leads to the secretion of various SASP factors which can contribute to salivary gland dysfunction and fibrosis [34]. As with IL-1α and TNF, TIMP-1 secretion levels have been found to be elevated in blood under chronic inflammation [35]. Additionally, TIMP-1 has been reported to be strongly associated with radiation-induced lung fibrosis by the induction of macrophage and neutrophil infiltration in lung tissue [36]. KC and MIP-1α chemokines play a driving role in the inflammatory processes and wound healing by recruiting and activating various types of leukocytes and macrophages [29,37,38,39]. On the other hand, treatment with G-CSF, which is usually secreted in response to inflammatory stimuli, was found to increase the concentration of bone marrow-derived cells in irradiated salivary glands in mice and improve morphology and function of the salivary gland by reducing the loss of acinar cells [40].

Sjögren’s syndrome is an autoimmune disease characterized by progressive destruction and dysfunction of salivary glands in many respects similar to the late effects in salivary glands after irradiation. It is believed that dysregulation of cytokine signaling plays a role in both systemic and exocrine gland manifestations of Sjögren’s syndrome [24]. TNF levels in the blood and in lymphocytic infiltrates in salivary gland biopsies have been found to be increased in patients with Sjögren’s syndrome compared to controls [41], which was associated with salivary gland hypofunction caused by inflammation [42]. Experiments in mice that mimicked the inflammatory conditions of swollen salivary glands showed that conditional overexpression of TNF levels in salivary gland biopsies led to acinar cell atrophy and hyposalivation [42], which is consistent with our findings but in saliva samples. It was also found that the biopsies from the salivary glands of patients with Sjögren’s syndrome expressed higher concentrations of MIP-1α [43].

The cytokines detected in this work (IL-1α, TNF, G-CSF, KC, MIP-1α, and TIMP1) have been found in other mouse studies, though in plasma and tissue samples after single or fractionated photon or proton irradiation [15,17,18,19,20]. However, only one study in rats reported a specific cytokine profile including IL-1α, IL-2, IL-6, IL-10, TNF, IFN-γ, VEGF, and GM-CSF in association with late effects progressing after a 30 Gy single-dose irradiation [15]. In our study, we observed a positive correlation between all cytokines in the control group, while both positive and negative correlations were observed in the irradiated group. This most likely indicates that irradiation-induced processes have led to alterations in the regulation of cytokine secretion. The correlation matrices of salivary cytokines and the two endpoints showed a high mean correlation, although with different signs for the two endpoints (high degree of fibrotic area and low saliva volume indicate decreased salivary gland function). A much stronger correlation between the salivary cytokines with endpoints compared to the serum cytokines supported our hypothesis that serum cytokines are less specific and less sensitive to the local damage of salivary glands than salivary cytokines. Therefore, saliva has a higher potential to be a reliable biomarker of local radiation-induced salivary gland damage.

Cytokine biomarkers may contribute to predicting or monitoring the response of normal tissues following radiotherapy and can also be of potential use for individualized in vivo dosimetry for biologically adaptive radiotherapy [44]. In addition, the biomarker can be employed during treatment to identify the need for preventive treatment of normal tissue toxicities or adjustment of the radiotherapy schedule. However, early prediction of late radiation effects using a panel of cytokines with multiple linear regression may be hampered by multicollinearity and a high risk of overfitting. For identification of potential biomarkers of late effects, we used a combined late effect score including both fibrotic area and saliva volume in a concatenated manner. By this, we generated a more robust endpoint series, which is better suited for a machine learning algorithm such as decision tree regression. Also, we used leave-one-out cross-validation, holding out one animal from the model training in each step. A feature vector based on all cytokines at day 35 was generated. The model gave a clear separation between the irradiated group and the control group but worked better for the fibrotic area than the saliva volume. This is not surprising since there was a larger spread in the saliva volume measurements within the groups and a smaller difference between controls and irradiated animals for saliva volume than for fibrosis. Also, fibrosis within the gland may be more directly linked to irradiation, while hyposalivation may also occur due to various direct and indirect radiation responses such as glandular, ductal, and nerve damage, acinar atrophy, and fibrosis. Notably, the feature importance analysis showed that IL-1α levels on day 35 were by far the strongest contributor to the prediction model. In contrast, the prediction was poorer when cytokine expression at day 80 or 105 was used in the modeling. This indicates that IL-1α expression around day 35 is highly involved in the pathological processes leading to fibrosis and hyposalivation. Therefore, inhibition of IL-1α might be considered as potential intervention target to mitigate radiation-induced fibrosis, but more studies should be conducted to elucidate the mechanisms of cytokine regulation. Furthermore, in a human population, this time point might be different and must be identified using a similar methodology as in the current work.

## 4. Materials and Methods

The procedures, protocols, and set-up for local H&N irradiation of mice have previously been reported in detail [45].

### 4.1. Animals, Irradiation, and Follow-Up

C57BL/6J female mice from Janvier (France) were used. All experiments were performed in accordance with directive 2010/63/EU on the protection of animals used for scientific purposes and approved by the Norwegian Food Safety Authority (ID 27931). The animals were 12 weeks old at the onset of experiments.

X-ray treatment was given in 10 fractions over 5 days (twice a day with an 8 h difference) using a Faxitron Multirad225 irradiation system (Faxitron Bioptics, Tucson, AZ, USA) with the following settings: 100 kV X-ray voltage, 15 mA current, and 2.0 mm Al filter. The dose rate was 0.66 Gy/min. For irradiation, the mice were anesthetized using Sevoflurane 4% in O_2_, positioned on the right side in a foam holder, and irradiated with the X-ray beam coming from the left. The radiation field included the oral cavity, pharynx, and major salivary glands and was defined by the lead collimator with a 25 × 20 mm opening to avoid the exposure of the eyes and brain.

**Figure 6:**
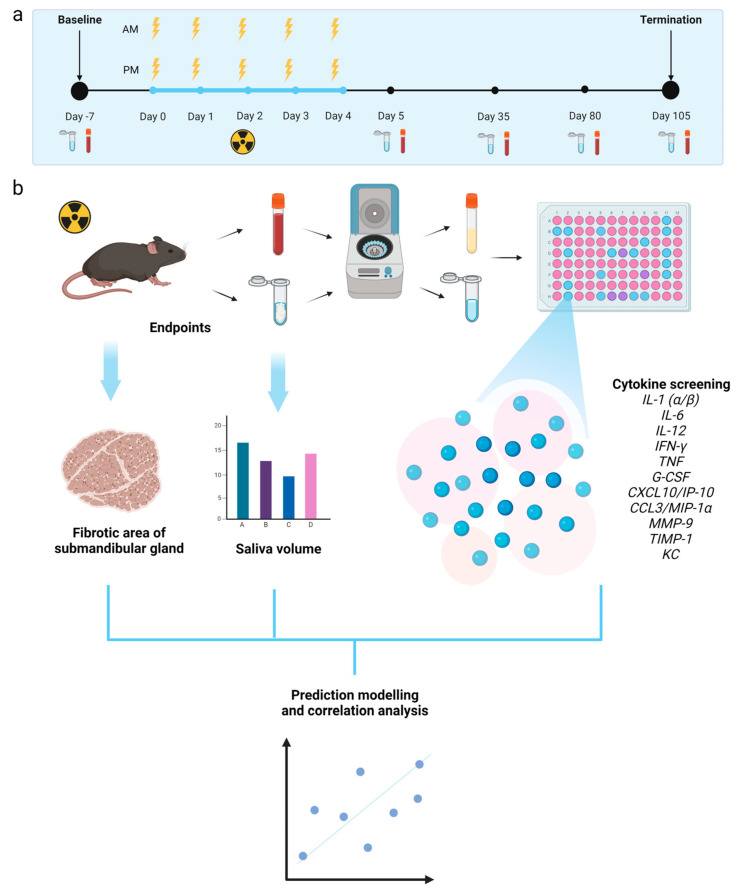
(**a**) Timeline of the experiment. (**b**) General overview of data collection and processing. Created with BioRender.com (accessed on 6 October 2023).

Twenty female mice were randomly assigned to either sham treatment or 10 × 6.6 Gy (*n* = 10 for each treatment group). The reported tissue dose is the mean dose calculated at the midpoint of the X-ray path through the mouse. The experimental timeline with data collection and processing overview is presented in Figure 6. On day −7, blood and saliva sampling were performed in all animals as baseline measurements. On days 0–4, fractionated treatment was given twice a day to the irradiation group, as explained above. Additional blood and saliva samplings were performed on days 5 (the day after the completion of irradiation), 35, 80, and 105 before termination. Blood and saliva samples were used for cytokine analysis (see below). The follow-up period in this study was 105 days to encompass the manifestation of both early and late radiation-induced tissue effects. During the follow-up period, the animal appearance (out-field), body weight loss (max 20% from baseline), and skin/oral mucosa (in-field) scores were used as humane endpoints. Upon the development of moderate to severe early effects, DietGel^®^ (ClearH_2_0, Westbrook, ME, USA) was provided to support the recovery, and analgesic treatment of buprenorphine (Temgesic, Indivior, Richmond, VA, USA) by subcutaneous injections was given to all mice for 4 days (day 12 to day 15, first injection after blood and saliva sampling) to alleviate the pain.

Mice were euthanized through an overdose of anesthetic (Pentobarbitol, Exagon^®^ Vet, Richter Pharma AG, Wels, Austria) by intraperitoneal injection under terminal anesthesia to avoid H&N tissue damage from cervical dislocation. The left submandibular gland (SMG) in each animal was harvested and fixed in 10% formalin before undergoing dehydration and paraffin embedding. The SMG was used to quantify radiation-induced fibrosis since this gland is the largest salivary gland in mice with a mixed serous and mucous secretion. Saliva volume and fibrotic area of SMG were used as endpoints of late effects in this study.

### 4.2. Blood and Saliva Sampling

Blood from the tail vein was collected into serum microvette tubes (Microvette, Sarstedt). The samples were centrifuged at 1000× *g* and 4 °C for 15 min, and the separated serum was stored at −80 °C. Saliva collection was performed as previously described [46]. Briefly, 0.375 mg/kg of pilocarpine (Pilocarpine hydrochloride, Sigma-Aldrich Inc., St. Louis, MO, USA) was intraperitoneally administered to the mice under anesthesia to stimulate saliva production. Saliva was collected into a cotton swab for 15 min, which was then centrifuged at 7500× *g* and 4 °C for 2 min. The obtained volume was measured and stored at −80 °C until cytokine analysis. For saliva volume estimates, data from days 80 and 105 were pooled because of inter-animal variations due to the limited number of animals per group.

### 4.3. Cytokine and Chemokine Analysis

Serum and saliva samples were thawed on ice, vortexed, and spun down at 16,000× *g* for 5 min at 4 °C. The samples were diluted (serum 1:1 and saliva 2:1) with the RD1-W buffer (R&D Systems, Abington, UK). All samples were analyzed with the custom-made 12-plex Luminex Mouse Discovery Assay kit (http://www.biotechne.com/g8AnddcM (accessed on 31 March 2022)) including CCL3/MIP-1α, KC, IP-10, G-CSF, IFN-γ, IL-1α, IL-1β, IL-6, IL-12 p70, MMP-9, TIMP-1, and TNF (Bio-Techne Ltd., Abington, UK). The plates with saliva samples were incubated overnight. A Luminex IS 200 instrument (Bio-Rad, Hercules, CA, USA) was used to record data. IL-1β, IL-6, IP-10, or IFN-γ were below the level of detection in saliva samples, while MMP-9 was excluded for both serum and saliva samples as the data were above the standard curve. Due to high background levels, IL-12 p70 was also excluded from the cytokine panel. Total protein concentrations in saliva samples were measured in mg/mL using spectrophotometry (Absorbance 280 nm, NanoDrop 2000c, Thermo Fisher Scientific, Waltham, MA, USA). The salivary cytokine levels were adjusted to total protein concentration and presented as (pg of cytokine)/(mg of total protein).

### 4.4. Quantitative Analysis of Fibrosis

A central section (6 μm) of each SMG was stained with Masson Trichrome (Trichrome Stain kit, abcam) according to the protocol from the manufacturer. Images of the stained sections were acquired using a Nikon DS-Ri1 camera with a CFI Plan Fluor 10× objective (NA 0.30). The percentage of fibrotic area (blue area in Figure 3a) relative to the total area was extracted by thresholding in ImageJ based on 8–11 images of the SMG from each mouse. One irradiated SMG was excluded as the stained section did not encompass the entire gland tissue. The percentage of fibrotic area in each SMG was termed the fibrosis score.

### 4.5. Prediction of Late Effects Based on Cytokine Levels

Python (v3.9.13) together with scikit-learn [47] was used to investigate whether cytokine levels could predict late effects in terms of hyposalivation or fibrosis. A per-day feature matrix was generated, consisting of cytokine levels per mouse converted to a standard score with a mean of zero and a standard deviation of unity for each cytokine. An endpoint vector was generated by concatenating the standard score vector for fibrotic area and saliva volume. The saliva volume vector was negated in order to align this score with that of the fibrotic area (a high value means a worse outcome). Decision tree regression with three nodes was used with the feature matrix as the dependent variable and the concatenated endpoint vector as the independent variable. Leave-one-out-cross-validation with the calculation of root-mean-square error (RMSE) was used to evaluate the prediction performance. The contribution of each cytokine to the final model was evaluated in terms of Breiman’s feature importance metric.

### 4.6. Statistical Analysis

Statistical analysis was performed using Prism 8 for Windows (Version 8.3.0, GraphPad Software, Boston, MA, USA) and Python 3.9.13. A significance level of 0.05 was used for all analyses. Pearson’s correlation was used to measure the strength and direction of a linear relationship between cytokine concentrations and/or endpoints.

## 5. Conclusions

To our knowledge, this is the first study monitoring both salivary and serum cytokines after H&N irradiation. A differential secretion of cytokines was detected in saliva from irradiated mice compared to the controls. The majority of the investigated salivary cytokines showed increased levels from day 35 after irradiation. A strong correlation between salivary cytokine levels and late endpoints was found, while cytokines from serum were only weakly correlated with the endpoints. Decision tree regression identified IL-1α as the strongest contributor with respect to the prediction of late endpoints, indicating that this cytokine might be a key in the pathological processes leading to fibrosis and hyposalivation.

## Data Availability

The data presented in this study are available on request from the corresponding author.

## Supplementary Materials

The following supporting information can be downloaded at: https://www.mdpi.com/article/10.3390/ijms242015218/s1.
