# Supplementary material for: Cytokine Levels in Saliva Are Associated with Salivary Gland Fibrosis and Hyposalivation in Mice after Fractionated Radiotherapy of the Head and Neck
Source: Int J Mol Sci. 2023 Oct 16;24(20):15218. doi: 10.3390/ijms242015218 (PMC10607825; doi:10.3390/ijms242015218)
Supplement: Supplementary file 1 [file ijms-24-15218-s001.zip › ijms-2591732-supplementary.pdf]

Cytokine levels in saliva are associated with salivary gland fibrosis and hyposalivation in mice after fractionated radiotherapy of the head and neck

Supplementary figures

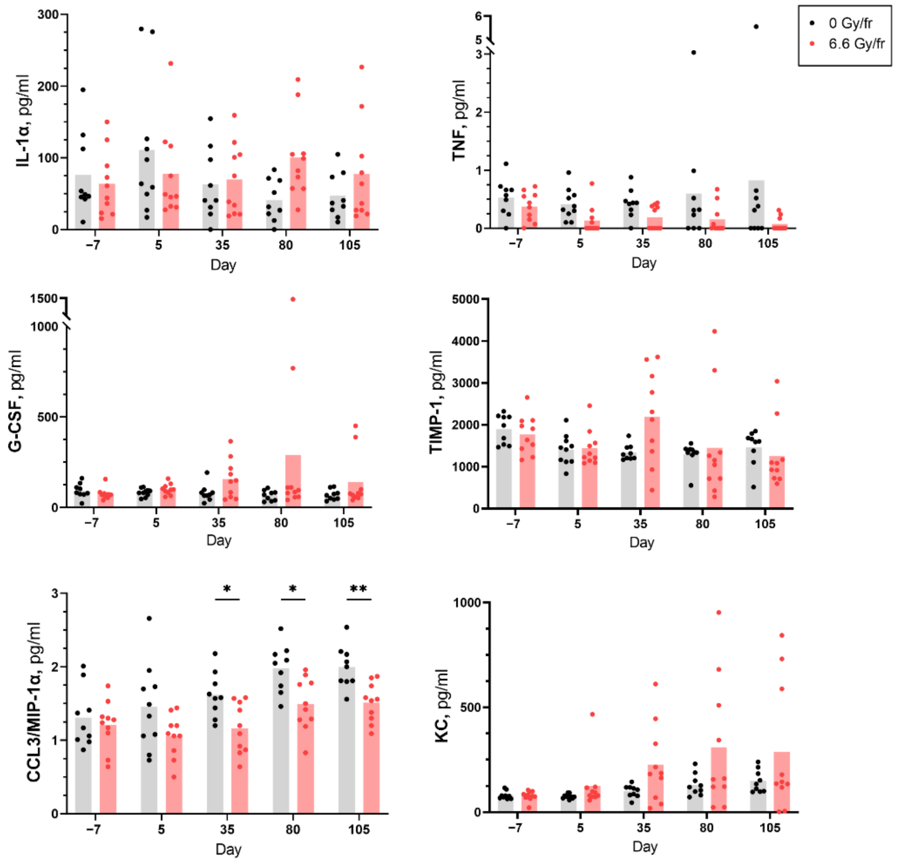

Figure S1. Levels of serum cytokines IL-1α, TNF, G-CSF, TIMP-1, MIP-1α and KC before and after fractionated irradiation at days -7, 5, 35, 80 and 105 (n = 9 in the control group, n = 10 in the irradiated group). Each dot represents an individual mouse. Data are presented as mean pg/mL cytokine (*p<0.05, **p<0.01).

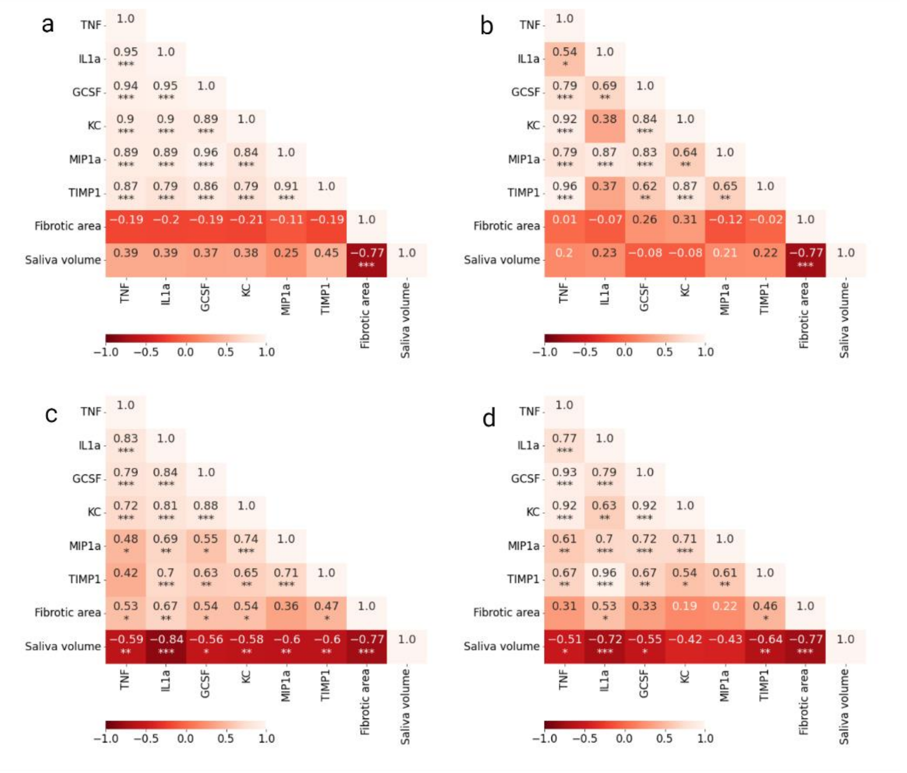

Figure S2. Pearson's correlation matrix with significance for combined (controls and irradiated) salivary cytokine expression on: (**a**) baseline, (**b**) day 5, (**c**) day 80, (**d**) day 105 (*p<0.05, **p<0.01, ***p<0.001).

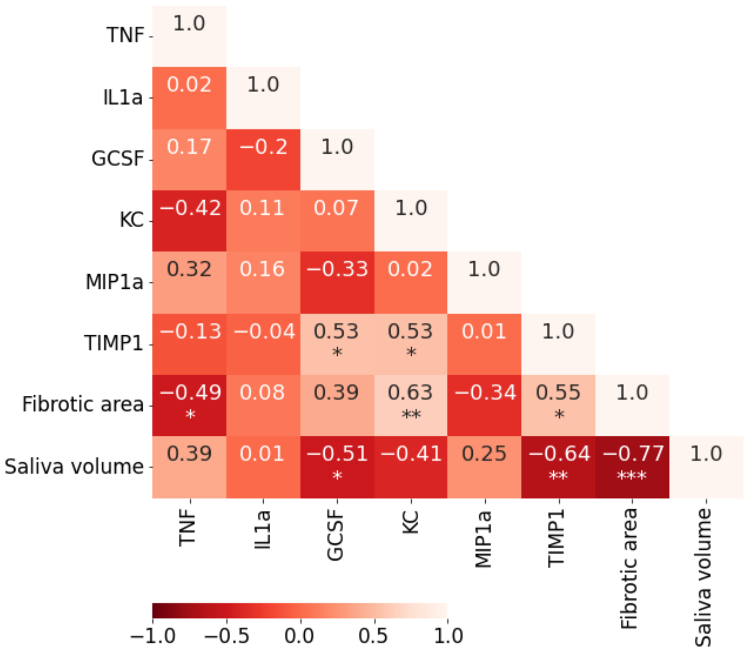

Figure S3. Pearson's correlation matrix with significance for combined cytokine levels in serum on day 35 (*p<0.05, **p<0.01, ***p<0.001).
